# Effect of Heat Treatment on the Structure and Properties of Silver-Coated Copper Powder

**DOI:** 10.3390/ma18050940

**Published:** 2025-02-21

**Authors:** Bingzhe Yang, Xiaoyun Zhu, Xiang Li, Junquan Chen, Nan Yang

**Affiliations:** 1 Faculty of Materials Science and Engineering, Kunming University of Science and Technology, Kunming 650093, China; yang_5413@163.com (B.Y.); c2298405268@163.com (J.C.); yn009612819@163.com (N.Y.); 2 Yunnan Spring New Material Co., Ltd., Kunming 650093, China

**Keywords:** silver-coated copper powder, heat treatment, electrical conductivity, surface morphology, antioxidant performance

## Abstract

Silver-coated copper powder, a cost-effective alternative to pure silver, has gained attention for its potential applications in electronics, energy, and catalysis. To explore the impact of heat treatment on its properties, a series of experiments were conducted with temperature increments of 50 °C and varying holding times. The silver-coated copper powder was prepared through chemical plating and was heat-treated to assess changes in surface morphology, conductivity, density, and antioxidant performance. Results show that heat treatment significantly improved surface flatness and smoothness, particularly at 600 °C for 5 min followed by 700 °C for 10 min. After treatment, the specific surface area decreased from 0.2282 m^2^/g to 0.2217 m^2^/g, while bulk density increased from 2.813 g/cm^3^ to 2.945 g/cm^3^, improving fluidity and stability. However, dislocation defects and the elimination of surface plasmon coupling between silver particles reduced conductivity, with resistance rising from 2.8 mΩ to 3.2 mΩ. X-ray diffraction showed an increase in silver grain size from 13.68 nm to 20.33 nm, enhancing density but increasing electron scattering. Heat treatment also raised the initial oxidation temperature from 200 °C to 230 °C but accelerated subsequent oxidation. This study improves upon existing technology by significantly enhancing the surface smoothness, oxidation resistance, density, and specific surface area of the silver-coated copper powder.

## 1. Introduction

Silver-based conductive materials, particularly those using silver particles as the main conductive phase, have attracted significant attention in high-tech industries such as electronics, energy, and catalysis due to their exceptional electrical and thermal conductivity, as well as their chemical stability [1,2,3,4,5,6]. Silver’s unparalleled performance makes it an ideal material for applications like conductive inks, electronic packaging, and catalytic converters. However, the rising cost and limited availability of silver present substantial challenges for its widespread industrial use [7]. The increasing prices of silver have created a growing demand for cost-effective alternatives that retain silver’s desirable properties while reducing consumption. In this context, silver-coated copper powder has emerged as a promising substitute for pure silver powder, offering a balance between performance and cost-efficiency. By combining silver’s high conductivity with copper’s lower cost, this material proves well-suited for applications in low-temperature silver pastes, conductive coatings, and other related fields.

The development of silver-coated copper powder has been widely explored in recent years, with several preparation methods being developed, including spray pyrolysis [8,9], electron beam radiation [10], vapor-phase spark ablation [11], and chemical plating [12,13]. Spray pyrolysis involves spraying a solution containing copper and silver precursors into a high-temperature furnace, where the precursors decompose to form silver-coated copper particles [8,9]. While this method can produce uniform coatings, it requires high temperatures and complex equipment, resulting in higher production costs. Electron beam radiation uses electron beams to induce the reduction of silver ions onto copper particles [10]. Although this process can yield high-quality coatings, its need for specialized equipment and high energy consumption limits its industrial feasibility. Vapor-phase spark ablation ablates a metal target in a vapor phase, leading to the formation of nanoparticles [11]. While it provides high purity and uniform coatings, it is still experimental and unsuitable for large-scale production. In contrast, chemical plating has emerged as the most practical method for the large-scale production of silver-coated copper powder due to its simplicity, low cost, and ability to produce uniform coatings [12,13]. Previous studies have examined the growth mechanisms and surface characteristics of silver coatings on copper particles. For example, Wan et al. [14] used first-principles calculations to demonstrate that silver growth on copper surfaces follows the Stranski–Krastanow heterogeneous growth model, which inevitably leads to surface roughness and protrusions. Similarly, Choi et al. [15] synthesized submicron-sized silver-coated copper particles but faced challenges in achieving uniform surface smoothness. These findings highlight the need for further optimization of the surface quality of silver-coated copper powder to enhance its performance in practical applications.

Heat treatment is a widely employed technique for modifying the microstructure and properties of materials by adjusting parameters such as temperature, atmosphere, and time. It has proven effective in altering surface morphology, particle size, and distribution, which in turn influences overall material performance [16]. In the case of silver-coated copper powder, heat treatment has the potential to improve surface smoothness, enhance density, and optimize electrical conductivity while also preserving oxidation resistance. For instance, Hai et al. [16] observed significant void formation on the surface of silver-coated copper powder at 500 °C but noted no significant improvement in surface roughness at temperatures below this threshold. This indicates that while structural changes begin at 500 °C, they are insufficient to optimize the powder’s properties. Therefore, exploring higher temperatures becomes necessary to achieve the desired improvements. Heat treatment can significantly alter the surface morphology of silver-coated copper powder. At higher temperatures, the silver layer undergoes structural reorganization, leading to a smoother surface, which is particularly beneficial for applications where surface roughness impacts performance, such as in conductive coatings. Additionally, heat treatment influences particle size and density. By optimizing temperature and holding time, it is possible to achieve a denser and more uniform powder, thereby enhancing its mechanical and electrical properties. One of the key advantages of silver-coated copper powder is its improved oxidation resistance compared to pure copper. Heat treatment can further enhance this property by forming a more stable silver layer, which is crucial for applications where the material is exposed to high temperatures and oxidative environments.

The goal of this study is to explore how heat treatment influences the structural and functional properties of silver-coated copper powder, ultimately aiming to optimize its performance for industrial applications. By examining changes in surface morphology, grain size, electrical conductivity, density, and oxidation resistance, we seek to identify the most effective heat treatment conditions that enhance the material’s properties while remaining cost-effective. This research intends to contribute to the advancement of silver-coated copper powder as a high-performance, cost-efficient alternative to pure silver in various technological applications.

The organization of this paper is as follows: Section 1 introduces the background and significance of the study. Section 2 describes the experimental methods used to prepare and heat-treat the silver-coated copper powder. Section 3 presents the results and discussion, detailing the effects of heat treatment on the structure and properties of the powder. Finally, Section 4 concludes the study with a summary of the key findings and their implications.

## 2. Materials and Methods

### 2.1. Sample Preparation

Copper powder pretreatment: A certain amount of copper powder was weighed, ultrasonic cleaning was performed with ethanol and hydrochloric acid to remove impurities and the surface oxide layer, and then the copper powder was thoroughly washed with deionized water for later use.

Chemical silver plating: A silver amine complex solution was prepared by mixing silver nitrate with triethanolamine. The pretreated copper powder was then added to deionized water and subjected to ultrasonic stirring for 20 min. Subsequently, the silver amine complex solution and ascorbic acid solution were added dropwise at a constant rate and allowed to react for 20 min, resulting in silver-plated copper powder. The experimental parameters are listed in Table 1.

### 2.2. Heat Treatment Method of Silver-Coated Copper Powder

The heat treatment of the silver-plated copper powder was carried out in a tube furnace under the experimental conditions outlined in Table 2. These conditions ensured a stable and controlled environment, preventing oxidation and improving the reproducibility of the results.

Hai et al. [16] observed significant void formation on the surface of silver-coated copper powder at 500 °C, with no notable improvement in surface roughness below this temperature. This suggests that while structural changes begin at 500 °C, they are insufficient for optimizing the powder’s properties. Based on this, 550 °C was selected as the lowest heat treatment temperature to assess the effects of higher temperatures while staying well below the melting points of silver (961.8 °C) and copper (1085 °C).

To systematically evaluate the influence of heat treatment, seven conditions were designed with a 50 °C increment, as shown in Table 3. The selected temperatures—550 °C, 600 °C, 650 °C, and 700 °C—cover a range that allows for a detailed analysis of how increasing temperature affects the powder’s structure and properties.

### 2.3. Characterization of Silver-Coated Copper Powder

The surface morphology and cross-section of the silver-coated copper powder were examined using a scanning electron microscope (SEM, XL30ESEM-TMP, Waltham, MA, USA). SEM is an effective technique for capturing high-resolution images of material surfaces. It works by scanning a focused electron beam across the sample, causing the material to emit secondary electrons. These electrons are then detected and used to generate an image, revealing detailed surface features and particle distribution [17]. The SEM in this study operates with an accelerating voltage of 15 kV and a working distance of 10 mm, offering magnification from 10× to 300,000× and a resolution of 1.2 nm at 15 kV. Measurements were conducted in a vacuum environment to ensure optimal imaging accuracy.

The specific surface area and pore size distribution of the silver-coated copper powder were measured using a surface area and pore size analyzer (ASAP 2460, Micromeritics Instrument Corporation, Norcross, GA, USA). This instrument utilizes the Brunauer–Emmett–Teller (BET) method, which involves nitrogen adsorption to determine the specific surface area of the sample [18]. The measurements were performed at a temperature of −196 °C (liquid nitrogen) and within a relative pressure range of 0.01 to 0.99. The surface area is measured within a range of 0.1 m^2^/g to 5000 m^2^/g with an accuracy of ±1%.

The elemental composition and distribution of the silver-coated copper powder were analyzed using an energy-dispersive X-ray spectrometer (EDS). EDS is a non-destructive technique that identifies and quantifies the elemental composition of materials. It operates by directing a focused electron beam onto the sample, which causes the atoms within the material to emit characteristic X-rays. Each element emits X-rays at distinct energy levels, allowing for precise identification. By analyzing these emitted X-rays, EDS can determine both the presence and relative abundance of elements in the sample [19]. The EDS system used in this study can detect elements from beryllium (Be) to uranium (U) with a detection limit of 0.1 wt%. Measurements were performed at an accelerating voltage of 20 kV and a working distance of 15 mm in a vacuum environment.

The crystal structure of the silver-coated copper powder was characterized using X-ray diffraction (XRD, D8A Advance, Bruker AXS, Karlsruhe, Germany). XRD analyzes the diffraction of X-rays by the crystalline structure of the sample, producing a diffraction pattern with peaks corresponding to specific crystal planes. These peaks can be compared to standard reference databases to identify the phases present in the material. The measurements were performed using a Cu Kα X-ray source (λ = 1.5406 Å) at an operating voltage of 40 kV and a current of 40 mA, with a scanning range of 20° to 80° (2θ). The detection range is 0.1° to 170° (2θ), and the system offers a resolution of 0.02°.

The optical properties, specifically the surface plasmon resonance, were analyzed using a UV-Vis spectrophotometer (UV-3600 Plus, Shimadzu Corporation, Kyoto, Japan). UV-Vis spectroscopy measures the absorption of ultraviolet and visible light by the sample, generating an absorption spectrum that reveals peaks at specific wavelengths. This technique is particularly effective for investigating the optical characteristics of materials and identifying surface plasmon resonance [20]. The measurements were conducted over a wavelength range of 200 nm to 800 nm with a resolution of 1 nm. The instrument’s detection range extends from 190 nm to 1100 nm with an accuracy of ±0.5%.

The thermal stability and oxidation resistance were evaluated using a thermal analyzer (TGA, STA 449 F3, NETZSCH Instruments, Selb, Germany) in air, with a heating rate of 10 °C/min. TGA measures the change in the mass of the sample as it is heated, providing insights into thermal decomposition and oxidation behaviors [21]. The instrument operates within a temperature range of −150 °C to 1600 °C with an accuracy of ±0.1 °C.

The electrical conductivity was measured using a digital ohmmeter (SD2002, Shenzhen Sunyage Electronic Co., Ltd., Shenzhen, China). This instrument measures the electrical resistance of the sample, with resistance inversely proportional to conductivity [22]. The measurements were performed at room temperature to ensure reliable results.

## 3. Results and Discussion

The properties of the silver-coated copper powder used in the experiment are summarized in Table 4.

### 3.1. Coating Morphology Analysis

**Figure 1:**
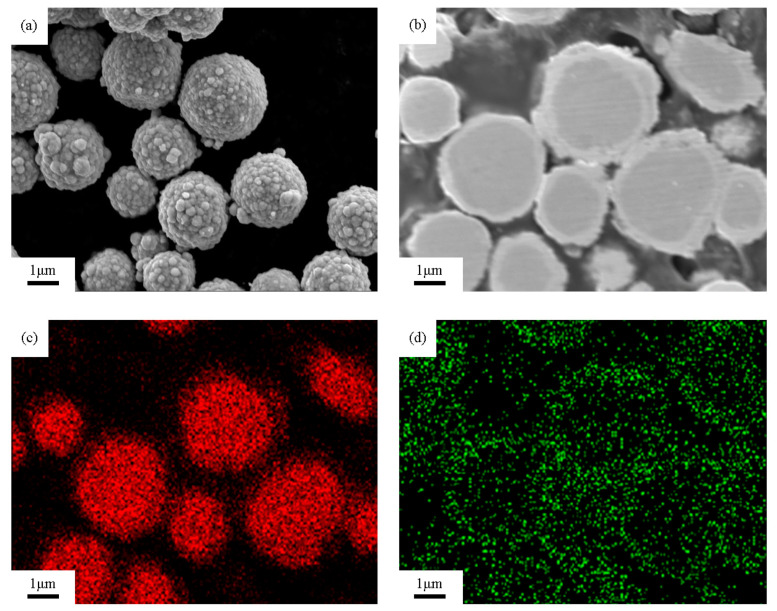
SEM images of silver-coated copper powder. (**a**) Secondary electron morphology, (**b**) cross-sectional secondary electron image, (**c**) energy spectrum mapping image corresponding to the Cu element, (**d**) energy spectrum mapping image corresponding to the Ag element.

The SEM morphology image and EDS composition distribution of the untreated silver-coated copper powder are shown in Figure 1. As seen in Figure 1a, the surface of the silver-coated copper powder, prepared by chemical silver plating, is covered with numerous nano-scale silver particles, forming a continuous and dense silver layer. However, the surface lacks uniform flatness. Figure 1b provides a cross-sectional view of Figure 1a, confirming that the silver coating is both continuous and dense, with an average thickness of about 470 nm. The corresponding energy spectrum mapping images for the copper and silver elements, shown in Figure 1c and Figure 1d, respectively, reveal that the silver coating on the copper substrate is continuous. However, the silver particles exhibit uneven size distribution, and the coating thickness is not uniform.

### 3.2. Effect of Holding Temperature on Silver-Coated Copper Powder

**Figure 2:**
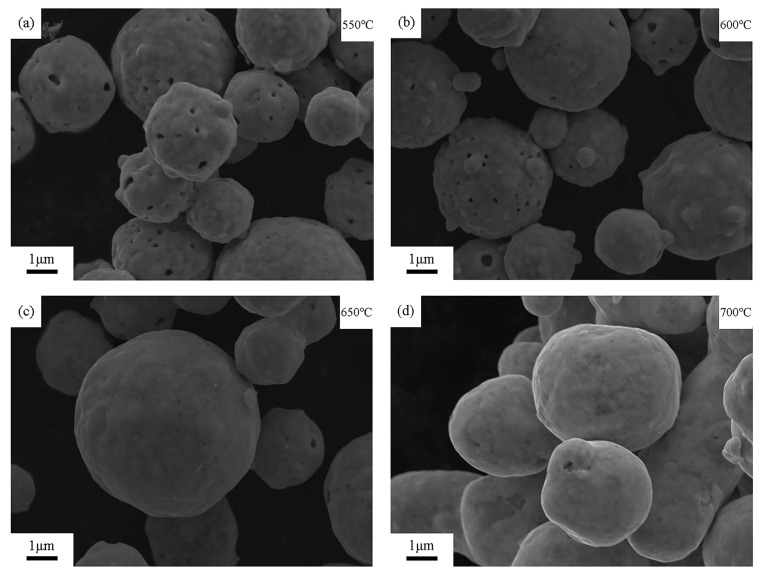
SEM images of the surface of silver-coated copper powder at heat treatment conditions of (**a**) 550 °C, (**b**) 600 °C, (**c**) 650 °C, and (**d**) 700 °C.

Figure 2 shows the surface SEM images of silver-coated copper powder heated at 550 °C, 600 °C, 650 °C, and 700 °C for 5 min. As seen in Figure 2a,b, at lower heat treatment temperatures, numerous holes appear on the surface of the silver-coated copper powder. These holes are believed to result from lattice mismatch and the release of strain energy from the silver particles during the deposition process. During the preparation of silver-coated copper powder, silver is deposited onto the copper surface in the form of small particles [14]. As the deposition occurs, the strain energy caused by lattice mismatch at the interface leads to the formation of irregular protrusions. For spherical particles, the formula for calculating surface energy γ is given in Equation (1) [23]:

(1) $$\gamma =\frac{3M}{r^{2}}$$

where M represents the molar mass of the particle and r is the particle radius. From this formula, it can be seen that as the particle radius (r) decreases, the surface free energy (γ) increases. During the heating process, particles with higher surface free energy will preferentially undergo structural reorganization. Since the mutual solubility of nano-scale Ag and Cu is low below 800 °C [24], the silver layer tends to agglomerate or dewet on the surface of the copper particles [16]. As the temperature rises, to reduce the overall surface free energy, the small Ag particles on the surface will spontaneously migrate and aggregate to form larger island structures. This leads to the formation of holes where the original small Ag particles were located. As the temperature continues to increase, in order to further lower the surface energy, the island structure transitions into liquid silver, which has fluidity. The liquid silver gradually fills the holes, ultimately smoothing the powder surface. As shown in the figure, when the heat treatment temperature reaches 650 °C, the holes on the powder surface show significant closure compared to treatments at 550 °C and 600 °C, leaving only a few small pores. The figure also indicates that after heat treatment at 700 °C, the pores on the surface of the silver-coated copper powder are completely closed, and the surface smoothness and flatness are notably improved compared to the untreated silver-coated copper powder.

**Figure 3:**
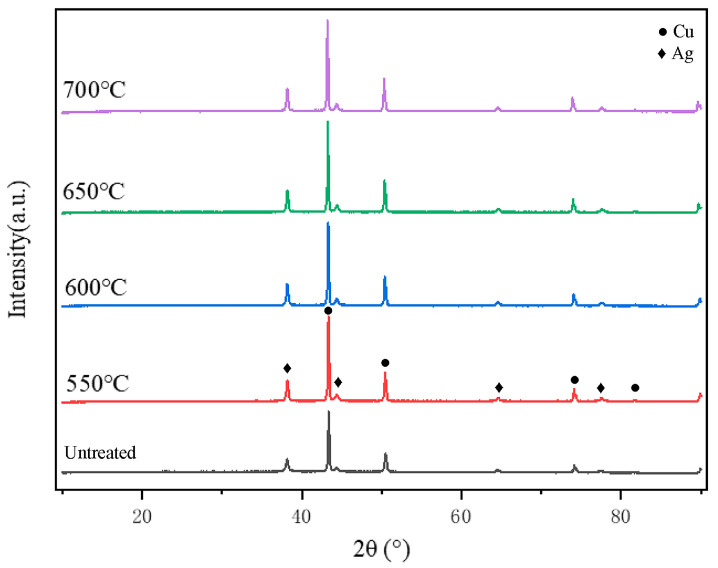
XRD patterns of silver-coated copper powder at different holding temperatures.

Figure 3 shows the X-ray diffraction (XRD) patterns at different holding temperatures with a holding time of 5 min. The analysis reveals that all four samples display eight distinct diffraction peaks. By comparing these peaks with the standard powder diffraction files (PDFs), they were confirmed to correspond to the physical phases of silver (Ag, PDF#04-0783) and copper (Cu, PDF#04-0836). Specifically, the diffraction angles (2θ) for the four characteristic peaks of silver are 38.116°, 44.277°, 64.426°, and 77.472°, corresponding to the (111), (200), (220), and (311) crystal planes of silver, respectively, consistent with the face-centered cubic structure of elemental silver. The diffraction angles (2θ) for the four characteristic peaks of copper are 43.297°, 50.433°, 74.130°, and 89.931°, corresponding to the (111), (200), (220), and (311) crystal planes of copper, which align with the face-centered cubic structure of elemental copper. The XRD analysis indicates that the heat treatment process does not cause any phase change in the silver-plated layer or the copper core, and both remain in their pure forms (though possible diffusion at the silver/copper interface cannot be excluded). Therefore, under the selected heat treatment conditions, the silver-coated copper powder remains stable throughout the process.

Heat treatment has a significant impact on grain size. The grain size can be calculated from the XRD data using the Scherrer equation [25]:

(2) $$D=\frac{K\lambda }{\beta \cos\theta }$$

where D represents the grain size, K is the Scherrer constant, λ is the X-ray wavelength, β is the full width at half maximum (FWHM) of the diffraction peak, and θ is the Bragg diffraction angle.

**Figure 4:**
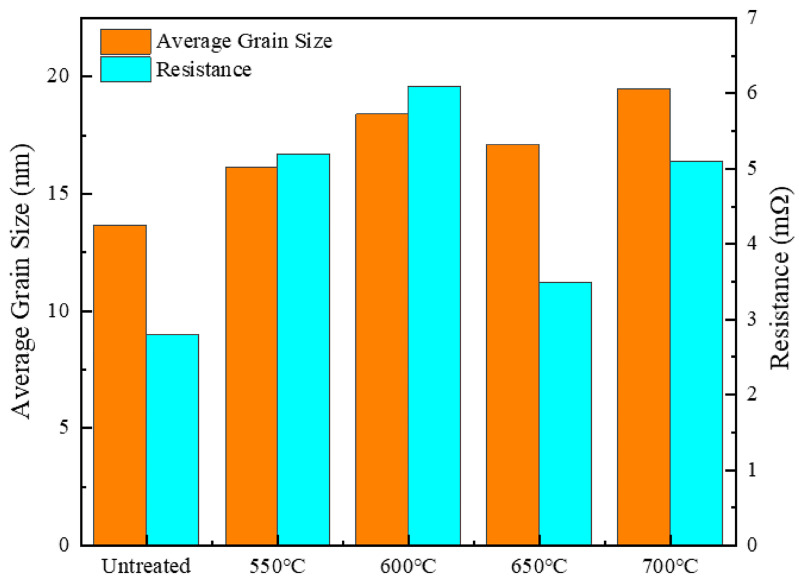
Average grain size and resistance of silver-coated copper powder at different holding temperatures.

Figure 4 shows the effect of different holding temperatures on the average grain size and resistance of silver-coated copper powder with a holding time of 5 min. After heat treatment, both the grain size and resistance of the silver-coated copper powder are higher compared to the untreated powder. The trends in the data are not straightforward, which can be attributed to the complex interactions of various factors during the heat treatment process. For grain size, from 550 °C to 600 °C, it increases by 14.06%. However, from 600 °C to 650 °C, it decreases by 6.98%, and from 650 °C to 700 °C, it increases again by 13.98%. Regarding resistance, from 550 °C to 600 °C, it increases by 17.31%, but from 600 °C to 650 °C, it decreases by 45.62% and from 650 °C to 700 °C, it increases by 45.71%. SEM analysis reveals that when the holding temperature is between 550 °C and 600 °C, the silver grain size increases, causing the silver layer on the powder’s surface to agglomerate. This results in the formation of numerous pores on the surface, leading to a decrease in the silver layer’s density and an increase in resistance. As the temperature rises, particularly when the holding temperature reaches 650 °C, the silver atoms rearrange, the grain size decreases, pores shrink, and the density of the silver layer increases, which lowers the resistance. However, as the temperature continues to rise to 700 °C, the grain size of the silver layer increases again, the density increases, and the resistance also rises. This indicates that during the grain growth process, lattice defects are formed in the silver layer, increasing electron scattering. This scattering hinders the effective transmission of electrons, ultimately reducing the conductivity of the silver-coated copper powder.

**Figure 5:**
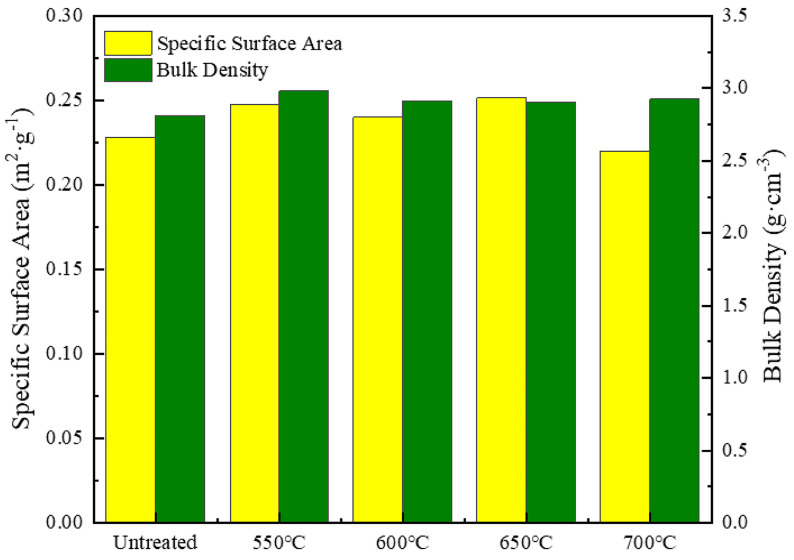
Specific surface area and bulk density of silver-coated copper powder at different holding temperatures.

Figure 5 shows how different heat treatment temperatures impact the specific surface area and bulk density of silver-coated copper powder. The specific surface area, which plays a crucial role in surface activity, flowability, adsorption, and the powder’s resistance to agglomeration and moisture [26,27], displays a complex trend with temperature. Between 550 °C and 600 °C, the specific surface area increased by 3.11%, likely due to the formation of additional surface pores, enhancing adsorption. From 600 °C to 650 °C, the surface area continued to increase slightly as pores connected, further boosting surface activity. However, between 650 °C and 700 °C, the specific surface area decreased significantly by 12.48%, suggesting that pores began to close, resulting in a smoother surface and reduced surface activity. This reduction in surface area also helps minimize particle attraction, reduces agglomeration, and improves the powder’s physical stability against environmental factors. Bulk density, another key indicator reflecting particle shape, size, surface roughness, and distribution, decreased by 2.25% from 550 °C to 600 °C due to the formation of island structures and pores. From 600 °C to 650 °C, bulk density remained relatively stable as the particle structure continued to adjust. However, from 650 °C to 700 °C, bulk density increased by 0.87%, driven by the disappearance of pores, increased sphericity, and reduced surface roughness, all of which improved flowability and stability. These percentage changes shed light on the complex trends observed, reflecting the interplay of pore formation, surface smoothing, and particle aggregation during heat treatment.

### 3.3. Effect of Holding Time on Silver-Coated Copper Powder

In this study, we chose 700 °C to examine the effect of holding time on the properties of silver-coated copper powder. This temperature was selected because it is close to the recrystallization temperature of silver (approximately 600–800 °C), where notable structural changes occur that can influence the material’s characteristics. Moreover, 700 °C is a commonly used temperature in heat treatment processes, making it an ideal condition for systematically investigating how holding time affects the material’s performance.

The 0 min hold time condition in the experimental design indicates that the powder is heated to 700 °C and then immediately cooled without remaining at this temperature for any duration. This is achieved by quickly heating the sample to 700 °C and then rapidly cooling it. This approach simulates rapid thermal treatment and allows us to observe the initial structural and property changes in the powder when exposed to high temperatures for a very short time.

**Figure 6:**
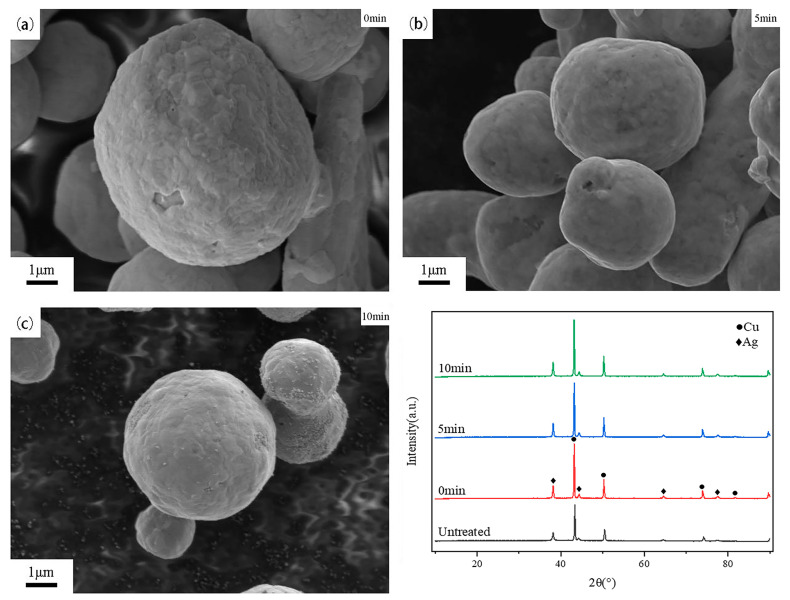
SEM images and XRD patterns of the silver-coated copper powder surface at a holding temperature of 700 °C for (**a**) 0 min, (**b**) 5 min, and (**c**) 10 min.

Figure 6 presents the SEM image of silver-coated copper powder held at 700 °C for 0, 5, and 10 min, along with the corresponding XRD patterns. As shown in the figure, at 0 min of holding time, the silver-coated copper powder does not form a distinct sintering neck. However, due to the agglomeration or dewetting of the silver layer, the silver coating on the powder surface becomes discontinuous. As a result, some copper particles remain uncovered, and the surface roughness is relatively high. As the holding time increases, the formation of sintering necks between the powder particles becomes more pronounced. This behavior is attributed to the thermal melting of the silver layer at 700 °C, which lowers the surface energy and enhances the mutual attraction between the particles. Consequently, the silver-coated copper powder particles tend to aggregate, forming larger agglomerates. The XRD patterns show that after heat treatment under all three conditions, the silver-coated copper powder remains in its elemental silver (Ag) and copper (Cu) forms.

**Figure 7:**
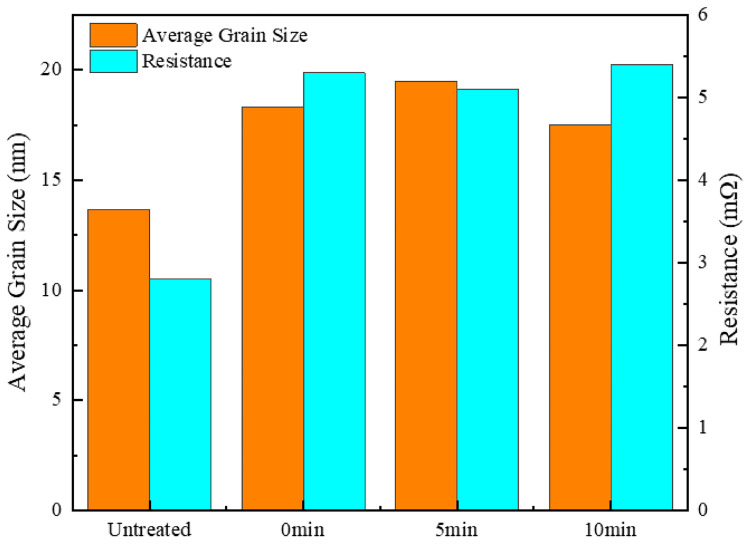
Average grain size and resistance of silver-coated copper powder at different holding times.

Figure 7 illustrates the impact of varying holding times at 700 °C on the average grain size and resistance of silver-coated copper powder. At 700 °C for 0 min, noticeable pores still appeared on the powder surface, resulting in a higher resistance compared to the silver-coated copper powder treated at 650 °C for 5 min. As the holding time increased to 5 min, the grain size of the silver layer grew by 40%, its density improved, and the resistance decreased by 3.77%. This initial increase in grain size and decrease in resistance can be attributed to the thermal melting and reorganization of the silver layer, leading to a denser and more conductive structure. However, as the holding time increased to 10 min, grain growth introduced lattice defects, causing electron scattering and increasing resistance. This led to a 5.88% increase in resistance. After 10 min, the grain size of the silver layer had increased by 28.57%, which reduced grain boundaries and consequently minimized the scattering effect at the boundaries. However, the increase in crystal defects due to the larger grain size led to a slight reduction in conductivity. As a result, the resistance of the silver-coated copper powder remained relatively stable under heat treatment conditions of 700 °C with holding times between 0 and 10 min.

**Figure 8:**
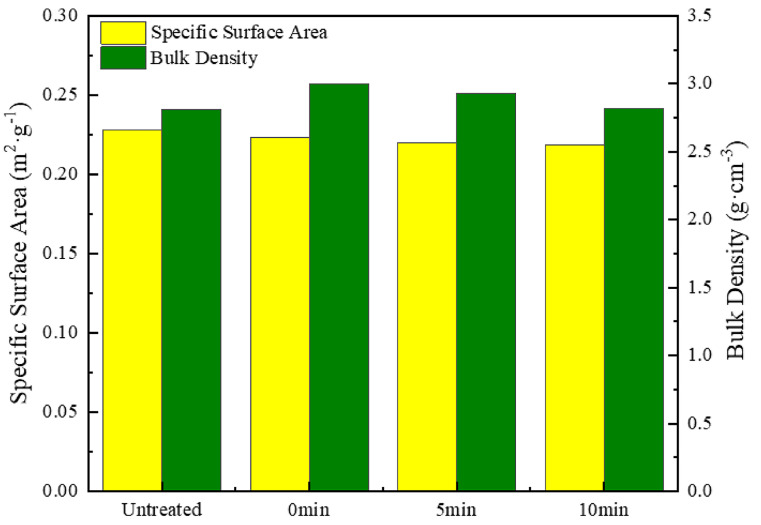
Specific surface area and bulk density of silver-coated copper powder at different holding times.

Figure 8 illustrates the impact of different holding times at 700 °C on the specific surface area and bulk density of silver-coated copper powder. As the holding time increases, the specific surface area decreases—falling by 1.52% from 0 to 5 min and by an additional 0.86% from 5 to 10 min. This reduction is primarily attributed to the decrease in surface pores and the formation of sintering necks between particles [28]. Similarly, the bulk density also decreases with increased holding time, dropping by 2.43% from 0 to 5 min and by 3.62% from 5 to 10 min. The decline in bulk density is likely due to the growth of sintering necks at high temperatures, which increases the gaps between particles and results in a reduction in overall bulk density.

### 3.4. Effect of Dual-Temperature Heat Treatment on Silver-Coated Copper Powder

The heat treatment condition H7 (600 °C for 5 min, followed by 700 °C for 0 min) represents a dual-temperature zone heat treatment. Table 5 presents a performance comparison between the silver-coated copper powder obtained under this heat treatment and the untreated silver-coated copper powder. The microstructure was examined using high-resolution transmission electron microscopy (HRTEM, FEI Talos F200S, Thermo Fisher Scientific, Waltham, MA, USA), while ultraviolet–visible absorption spectroscopy (UV-Vis) was used to analyze its antioxidant properties.

**Figure 9:**
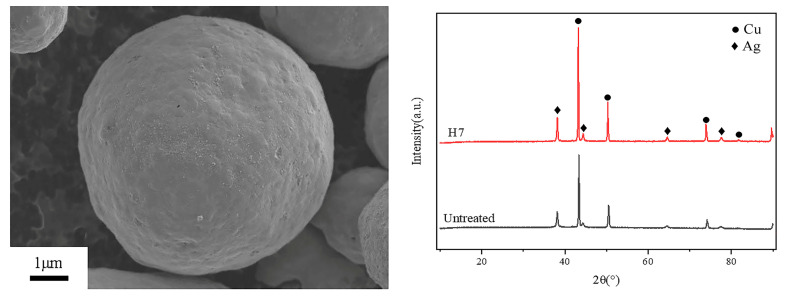
SEM image and XRD pattern of silver-coated copper powder under dual-temperature zone heat treatment condition H7 (600 °C for 5 min, heating to 700 °C for 0 min).

Figure 9 shows the silver-coated copper powder and XRD pattern obtained under heat treatment condition H7. Under these conditions, the surface of the powder exhibits high smoothness and flatness, with no apparent sintering necks between the particles. The 600 °C treatment for 5 min increases the surface energy of the silver layer on the copper powder. When heated to 700 °C, the silver layer melts and undergoes structural reorganization, resulting in a continuous and smooth silver coating. Moreover, the 0 min holding time at 700 °C effectively minimizes the time for the powders to attract each other after the silver layer melts, thereby reducing the formation of sintering necks between the particles. According to the XRD pattern, the silver-coated copper powder remains as elemental silver and copper after the dual-temperature zone heat treatment.

**Figure 10:**
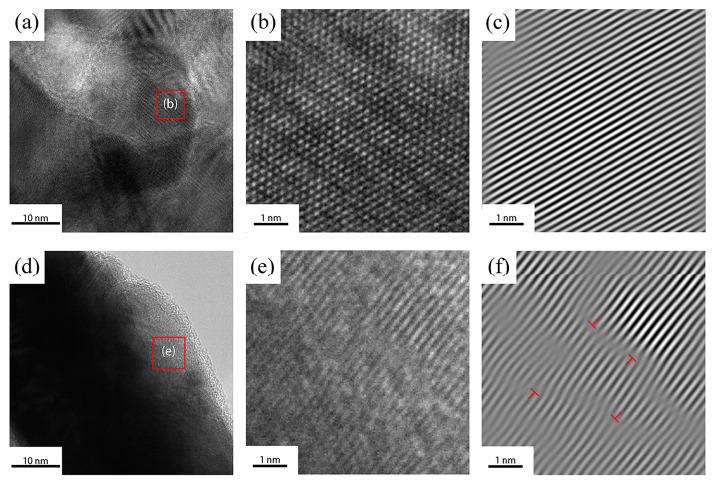
Silver-coated copper powder under heat treatment condition H0: (**a**) microstructure, (**b**) high-resolution transmission electron microscopy (HRTEM), (**c**) and inverse fast Fourier transform (IFFT); silver-coated copper powder under heat treatment condition H7 (600 °C for 5 min and then heated to 700 °C for 0 min): (**d**) microstructure, (**e**) high-resolution transmission electron microscopy (HRTEM), and (**f**) inverse fast Fourier transform (IFFT) (The red symbols are edge dislocation symbols).

Figure 10a shows the microstructure of the silver-rich region of the untreated silver-coated copper powder. Figure 10b presents the high-resolution transmission electron microscopy (HRTEM) image of the silver crystals in Figure 10a. Figure 10c displays the inverse fast Fourier transform (IFFT) result of Figure 10b. The lattice spacing in this region is 0.20125 nm, which is very close to the standard (2,0,0) lattice spacing of silver, which is 0.2043 nm. The slight deviation in the lattice spacing may be attributed to the fact that the silver layer is formed by numerous small nano-silver particles during the chemical plating process. These silver particles compress against each other during synthesis, leading to a slight alteration in the lattice spacing.

Figure 10d shows the microstructure of the silver-rich layer of the H7-treated silver-coated copper powder. Figure 10e presents the HRTEM image of the silver crystals from Figure 10d. Figure 10f displays the IFFT results of Figure 10e, revealing the presence of numerous dislocations in the silver layer after heat treatment. These dislocations increase electron scattering, obstruct the effective transmission of electrons, and result in a decrease in the conductivity of the silver-coated copper powder [29].

**Figure 11:**
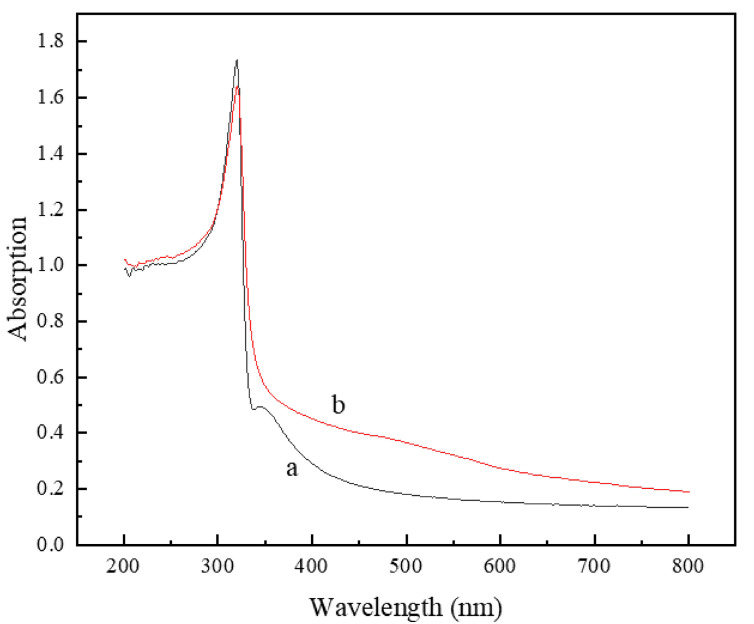
(a) UV absorption spectrum of silver-coated copper powder under heat treatment condition H0 and (b) UV absorption spectrum of silver-coated copper powder under heat treatment condition H7.

Figure 11 displays the UV–visible absorption spectra of the silver-coated copper powder both without heat treatment and under heat treatment condition H7. As shown in Figure 11a, the untreated silver-coated copper powder exhibits two distinct absorption peaks at 320 nm and 344 nm. The peak at 320 nm is associated with the single-particle resonance of individual silver particles, while the peak at 344 nm likely results from gap-coupling resonance between nanoparticles in silver nanoparticle dimers and trimers. This behavior aligns with the observation that the silver-coated copper powder, produced by chemical plating, is covered by a large number of silver nanoparticles with free silver present. In this scenario, the distances between different particles and between particles and free silver are extremely small, leading to strong coupling between surface plasmon waves, which influences the absorption characteristics [30]. Figure 11b presents the UV-Vis absorption spectrum of the H7 silver-coated copper powder after heat treatment. The spectrum shows a single absorption peak at 320 nm, which corresponds with the SEM observations that the surface of the silver-coated copper powder is now continuous, without any visible nanoparticles or free silver. This indicates that after heat treatment, the silver layer on the surface of the silver-coated copper powder no longer consists of dispersed silver particles but rather forms a continuous layer. The small free silver particles are either destroyed or absorbed during the structural reorganization process. However, the disappearance of the surface plasmon coupling structure between the nano-silver particles leads to a decrease in the conductivity of the silver-coated copper powder, which is consistent with the measured resistance data.

In summary, heat treatment induces significant structural changes in the silver layer of silver-coated copper powder, which contributes to the observed decrease in conductivity. The first key factor is the generation of dislocation defects. During heat treatment, thermal stress and grain growth lead to the formation of numerous dislocations within the silver layer. These dislocations act as centers for electron scattering, increasing electron scattering and hindering the efficient transmission of electrons, thus reducing conductivity. High-resolution transmission electron microscopy (HRTEM) images clearly reveal the presence of these dislocations (see Figure 10f). Secondly, changes in grain size play a significant role. Heat treatment causes the grain size of the silver layer to vary; between 600 °C and 650 °C, the grain size initially decreases and then increases. The increase in grain size enhances electron scattering and raises resistance. For instance, the sample held at 700 °C for 5 min exhibited a 40% increase in grain size, resulting in a 3.77% increase in resistance (see Figure 7). Thirdly, the disappearance of the surface plasmon coupling structure negatively impacts conductivity. In the untreated silver-coated copper powder, a large number of silver nanoparticles on the surface exhibit surface plasmon coupling, which enhances conductivity. However, after heat treatment, the silver layer forms a continuous film, and the surface plasmon coupling structure disappears, leading to a reduction in conductivity (see Figure 11). Collectively, these structural changes—dislocation defects, grain size alterations, and the loss of surface plasmon coupling—contribute to the decrease in conductivity of the silver-coated copper powder. As the temperature increases, the silver coating experiences thermal stress due to the temperature gradient and the differing thermal expansion coefficients of silver and copper. This stress leads to silver grains growing larger through Ostwald ripening, resulting in increased internal stress within the coating. When the stress exceeds the yield strength of the silver lattice, dislocations are generated. These dislocations, which are linear defects where the atomic arrangement is disrupted, can be observed in HRTEM images. Figure 10f shows a significant number of dislocations in the silver layer after heat treatment. These dislocations act as barriers to electron flow, increasing the resistance to electron migration. Dislocations serve as electron scattering centers, causing random electron deflections and disrupting their coherent flow. This scattering effect, known as electron–phonon scattering, significantly reduces electron mobility. As the density of dislocations increases, electron scattering becomes more pronounced, further lowering electron mobility and raising the material’s resistance.

**Figure 12:**
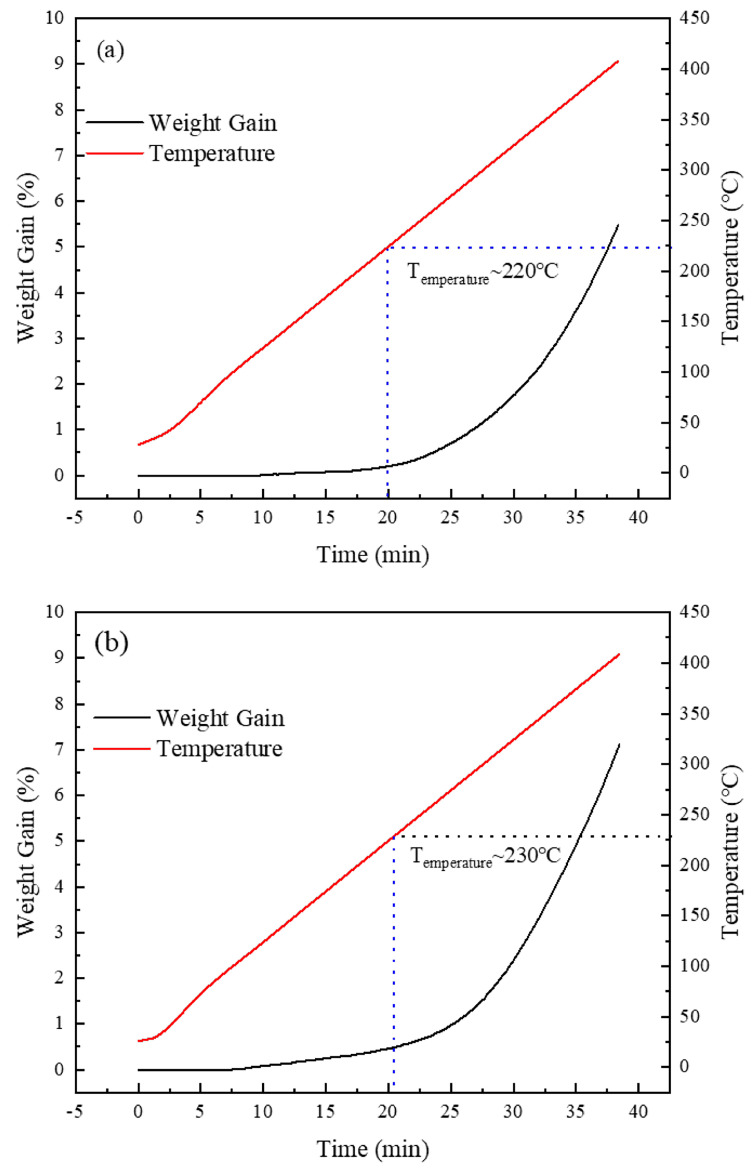
TGA spectra of silver-coated copper powder under different heat treatment conditions in air environment, (**a**) H0, (**b**) H7.

Figure 12 presents the thermogravimetric analysis (TGA) spectra of the silver-coated copper powder before and after heat treatment in air. The TGA results indicate that the silver-coated copper powder, with its dense silver coating, begins to oxidize when heated in air, a process typically linked to the dewetting of the silver layer. Oxidation generally starts around 200 °C [31,32,33]. Taking a weight gain of more than 0.2% as the threshold for the onset of oxidation, the data in Figure 7 show that the oxidation onset temperature of the silver-coated copper powder increases after heat treatment. This is consistent with the density increase observed in the XRD analysis. However, after the initial oxidation stage, the oxidation rate increases. This can be attributed to the enhanced surface energy and reactivity of the powder following heat treatment, which promotes oxygen molecule adsorption. Once the temperature reaches 230 °C, the silver layer begins to dewet, exposing the copper core to the air. The high surface energy and activity further accelerate the oxidation process, explaining the observed increase in the oxidation rate after the initial stage.

The increase in the initial oxidation temperature of silver-coated copper powder due to heat treatment holds significant benefits for both practical applications and long-term stability. The elevated oxidation onset temperature allows the material to endure higher temperatures before significant oxidation occurs, thus improving its thermal stability and reliability in high-temperature environments, such as electronic devices, conductive inks, and coatings. This is particularly advantageous in demanding applications like automotive electronics and aerospace components, where materials are exposed to elevated temperatures and oxidative conditions over extended periods. In terms of long-term stability, the delayed oxidation onset helps preserve the material’s electrical properties by protecting the copper core from oxidation, which can degrade performance. Although the oxidation rate increases after the initial phase, carefully controlled heat treatment conditions can optimize the balance between the initial oxidation temperature and the subsequent oxidation rate, enhancing the material’s stability throughout its service life. In summary, heat treatment strengthens the structural integrity of the silver coating, delays oxidation, and improves the long-term stability of silver-coated copper powder in various industrial and electronic applications.

Compared to single-temperature heat treatment, the double-temperature heat treatment (H7: 600 °C for 5 min, followed by 700 °C for 0 min) offers several advantages. Notably, it significantly enhances the surface flatness and smoothness of the silver-coated copper powder. As shown in the SEM image (Figure 9), this method creates a continuous uniform silver layer, effectively eliminating surface roughness and pores, with no noticeable sintering necks between particles. This indicates that the double-temperature treatment plays a key role in optimizing the microstructure of the silver-coated copper powder. Furthermore, it reduces powder agglomeration, improves stability, and lowers the specific surface area (from 0.2282 m^2^/g to 0.2217 m^2^/g). These changes suggest a reduction in surface activity and adsorption sites, which lowers particle interaction, improves fluidity, and prevents agglomeration. These benefits make silver-coated copper powder treated with a double-temperature approach more suitable for various industrial and electronic applications.

## 4. Conclusions

1. Surface morphology and smoothness: Heat treatment, particularly the dual-temperature method (600 °C for 5 min followed by 700 °C for 1 min), significantly enhanced the surface flatness and smoothness of the silver-coated copper powder. This process effectively removed surface roughness and pores, resulting in a continuous and uniform silver layer. As a result, the specific surface area decreased from 0.2282 m^2^/g to 0.2217 m^2^/g, while the bulk density increased from 2.813 g/cm^3^ to 2.945 g/cm^3^, leading to improved fluidity and stability.
2. Electrical conductivity: The elimination of dislocation defects and the loss of surface plasmon coupling between silver particles contributed to a decrease in electrical conductivity. As a result, the resistance increased from 2.8 mΩ to 3.2 mΩ. This reduction in conductivity can be attributed to the formation of dislocation defects and changes in grain size during heat treatment, both of which lead to increased electron scattering and hinder the efficient transport of electrons.
3. Oxidation resistance: The initial oxidation temperature increased from 200 °C to 230 °C, indicating an improvement in thermal stability. However, the heat treatment also raised the surface energy and reactivity, which led to an accelerated oxidation rate in the subsequent stages.
4. Overall performance and application: The experimental data in this study demonstrate that heat treatment significantly enhances the overall performance of silver-coated copper powder. These findings offer theoretical support for the material’s use in applications such as conductive inks, electronic packaging, and catalytic converters. Future research should focus on further optimizing the heat treatment conditions to achieve an optimal balance between conductivity and other enhanced properties, thereby maximizing the overall performance of silver-coated copper powder.

## Figures and Tables

**Table 1:** Silver plating composition and plating process parameters.

Ingredients	HCl/(mol·L^−1^)	Ag(NH_3_)^2+^/(mol·L^−1^)	VC/(mol·L^−1^)	pH	Temperature/(°C)	Dropping Speed/(mL·min^−1^)
Numeric	0.01	0.02	0.14	9–11	50	8–10

**Table 2:** Heat treatment experiment basic parameters.

Parameter	Atmosphere	Heating Speed/(°C·min^−1^)	Cooling Rate/(°C·min^−1^)	Nitrogen Flow Rate/(mL·min^−1^)	Internal Pressure/(kPa)
Numeric	Nitrogen	10	10	30	202.65

**Table 3:** Heat treatment conditions of experiments H1~H7.

	Heat Treatment Conditions
H0	Untreated
H1	Hold at 550 °C for 5 min
H2	Hold at 600 °C for 5 min
H3	Hold at 650 °C for 5 min
H4	Hold at 700 °C for 0 min
H5	Hold at 700 °C for 5 min
H6	Hold at 700 °C for 10 min
H7	Hold at 600 °C for 5 min, then heat to 700 °C and hold for 0 min

**Table 4:** Properties of silver-coated copper powder.

Property	Particle Size/(μm)	Resistance/(mΩ)	Specific Surface Area/(m^2^·g^−1^)	Bulk Density/(g·cm^−3^)	Theoreticsal Silver Content/(%)
Numeric	3–5	2.8	0.2282	2.813	15

**Table 5:** Properties of silver-coated copper powder prepared by heat treatment condition H7 and untreated silver-coated copper powder.

Property	Resistance/(mΩ)	Average Grain Size/(nm)	Specific Surface Area/(m^2^·g^−1^)	Bulk Density/(g·cm^−3^)
Untreated	2.8	13.68	0.2282	2.813
H7	3.2	20.33	0.2217	2.945

## Data Availability

The original contributions presented in this study are included in the article. Further inquiries can be directed to the corresponding authors.

## Abbreviations

The following abbreviations are used in this manuscript:

SEM	scanning electron microscope
EDS	energy-dispersive X-ray spectrometer
TEM	transmission electron microscope
XRD	X-ray diffraction
PDF	powder diffraction files
FWHM	full width at half maximum
HRTEM	high-resolution transmission electron microscopy
UV-Vis	ultraviolet–visible absorption spectroscopy
IFFT	inverse fast Fourier transform
TGA	thermogravimetric analysis
